# Serum Level of Matrix Metalloproteinase-9 in Patients with Salivary Gland Tumor

**Published:** 2014-12

**Authors:** Maryam Mardani, Azadeh Andisheh-Tadbir, Bijan Khademi, Peyman Biparva, Mahyar Malekzadeh

**Affiliations:** a Dept. of Oral Medicine, School of Dentistry, Shiraz University of Medical Sciences, Shiraz, Iran.; b Dept. of Oral and Maxillofacial Pathology, School of Dentistry, Shiraz University of Medical Sciences, Shiraz, Iran.; c Dept. of Otolaryngology, Khalili Hospital, Shiraz Institute for Cancer Research, Shiraz University of Medical Sciences, Shiraz, Iran.; d Undergraduate Student, Student Research Committee, School of Dentistry, Shiraz University of Medical Sciences, Shiraz, Iran.; e Shiraz Institute for Cancer Research, Shiraz University of Medical Sciences, Shiraz, Iran.

**Keywords:** Salivary gland tumor, Pleomorphic adenoma, Adenoid cystic carcinoma, Mucoepidermoidcarcinoma, Matrix Metalloproteinase 9

## Abstract

**Statement of the Problem:** Matrix metalloproteinases (MMPs) have been implicated in the pathogenesis of certain diseases and cancers via tissue destruction and can be secreted into the blood stream. MMP9 expression in the salivary gland tissue was evaluated but their serum level in the salivary gland tumors was not studied.

**Purpose:** The aim of our study was to determine the concentration of serum MMP-9 in healthy participants and in patients with salivary gland tumor.

**Materials and Method:** Using an ELISA kit, the circulating levels of MMP-9 in sera from 58 patients with salivary gland tumor (31 pleomorphic adenoma, 17 adenoid cystic carcinoma and 10 mucoepidermoid carcinoma) and 30 healthy controls was assessed.

**Results:** The serum MMP9 level in patients with salivary gland tumors (380.0±301.3 pg/ml) also patients with benign tumors (354.3±218.7 pg/ml) (354.3±218.7 pg/ml) were significantly lower than that in the healthy group (727.4±624.6 pg/ml) (Respectively *p*= 0.02 and *p*= 0.01). Mean serum MMP9 concentration in malignant tumors was (402.3±441.8pg/ml) higher than benign tumors (354.3±218.7 pg/ml) but the difference was not significant (*p*= 0.9).

**Conclusion:** Our results showed that serum level of MMP9 decreased in patients with salivary gland tumors which suggest that MMP9 may not have a potential role in development and pathogenesis of salivary gland tumor.

## Introduction

Carcinomas of the salivary glands constitute approximately 1 to 3% of all head and neck malignancies and 0.3% of all cancers.[1] They comprise a morphologically remarkable heterogeneous group of tumors. As a result of the morphological diversity of salivary gland tumors, the diagnosis and classification of them is a major challenge. There is also a great heterogeneity within the same tumor mass in addition to morphological variation among individual tumors.[2] The clinical behavior of salivary gland cancer and the responses to treatment vary in different histological types as well as within a particular histological entity, which may be associated with the biological heterogeneity of these tumors. These features confuse the use of prognostic tools when defining the aggressiveness of a malignant neoplasm.[3]

Matrix metalloproteinases (MMPs) are zinc-dependent endopeptidases which have a key role in the degradation of extracellular matrix.[4]

MMPs have an important role in the physiologic degradation of extracellular matrix (ECM) in the conditions like tissue repair, angiogenesis, bone resorption, tissue morphogenesis, and in the morphogenesis of branching epithelial organs such as salivary glands.[5] They also degrade several non-matrix proteins, comprising growth factors, chemokins, cytokins and their receptors so they can regulate cell growth and inflammation.[5]

MMPs can be classified into four groups: a) gelatinases b) collagenases c) stromelysins and d) membrane type MMPs.[6] Gelatinase B (MMP-9) is able to cleave the basement membrane collagens, which appear to be very critical in tumor cell invasion and penetration in the process of metastasis.[5]

Nagel et al. found that a disturbed balance exist between MMP and tissue inhibitor of metalloproteinase (TIMP) in malignant salivary gland tumors and suggested that MMP2 expression could be related to the invasive behavior and the malignant potential of these tumors.[7] De Vicente et al. proposed that the high expression of MMP9 could contribute to the prognosis and clinical behavior of malignant salivary gland tumors.[8]

MMP can be secreted into the blood stream; hence, it is assumed that MMP levels in the blood could serve as a biological marker for disease onset, progression, and monitoring in different cancers.[9-11] MMP9 expression in the salivary gland tissue was evaluated[7-8] but their serum level in the salivary gland tumors was not studied.

The aim of our study was to determine the concentration of serum MMP-9 in normal subjects and in the patients with salivary gland tumor. 

## Materials and Method

58 patients with salivary gland tumor (31 pleomorphic adenoma, 17 adenoid cystic carcinoma and 10 mucoepidermoid carcinoma) and 30 healthy control subjects were enrolled in this study. 

All the study patients were referred from ENT Department of Shiraz University of Medical Sciences and had been histopathologically diagnosed of salivary gland tumor.

Control cases were healthy blood donors who were matched the study group regarding age and gender. Exclusion criteria for both groups were the presence of any systemic disease, existence of periodontal disease, use of corticosteroid or non- steroid anti-inflammatory medication (at least during the last 3 months), or a history of malignancy of any type.

The Ethical committee of the Shiraz University of Medical Sciences approved the study and all the participants were informed about the nature of the study and agreed to participate by signing an informed consent form. 

Serum samples were obtained from clotted blood following the centrifugation of 4^o^C and storing at- 80^o^C until the time of analysis. MMP9 concentrations were measured by Sandwich ELISA following the manufacturer’s instructions (BMS; GmbH, Germany).

Statistical analysis was performed using the Mann-Whitney, Kruskal Wallis and Spearman correlation test. A *p*< 0.05 was considered as significant.

## Results

A total of 58 patients with salivary gland tumor (24 males and 34 females with mean age of 44.8±16.6 years) and 30 healthy control subjects (12 males and 18 females with mean age of 44.6±15.8 years) were enrolled in this study.

The serum MMP9 level in patients with salivary gland tumors (380.0±301.3 pg/ml) and also in patients with benign tumors (354.3±218.7 pg/ml) (354.3±218.7 pg/ml) were significantly lower than that in the healthy group (727.4±624.6 pg/ml) (*p*= 0.02 and *p*= 0.01 , respectively). Mean serum MMP9 concentration in malignant tumors was (402.3±441.8pg/ml) higher than benign tumors (354.3±218.7 pg/ml) but the difference was not statistically significant (*p*= 0.9). No relation was found between mean MMP9 levels and gender and age of the patients (respectively *p*= 0.4 and *p*= 0.9) and control groups (respectively *p*= 0.1 and *p*= 0.9).

## Discussion

The proteolytic cleavage of the basement membrane, a meshwork mainly composed of type IV collagen, is a critical step required for cancer metastasis.[8] Recent attention has been toward the MMP9 because it has been implicated to have a contributing role in cancer invasion and metastasis and hence the development of MMP inhibitors as antimetastatic therapy has been going ahead.[11-14]

MMP9 expression in salivary gland cancer has been studied briefly.[7-8, 15] De Vicente et al. showed that MMP9 protein was expressed in 63% of high grade salivary gland cancers and was significantly associated with regional and distant metastasis and consequently with the clinical stage of the disease. Therefore, it was proposed as a prognostic factor.[8]

Luukkaa et al. proposed that both MMP13 and MMP9 can promote the invasion of salivary gland cancer cells by enabling them to cleave basement membrane.[3]

The elevated serum or plasma levels of MMP9 were observed in patients with colorectal,[10] gastric,[16] lung,[17] and breast cancer[18] but decreased levels were seen in patients with bone tumors[19] and multiple myeloma [20] and some breast cancers.[21]

Since the serum is the most easily accessible and detectable body fluid, we have done the quantitative assay of circulating level of MMP9 in the sera of study group. In the present study, we showed that serum MMP9 levels in patients were significantly lower than that in the control group.

Two forms of pro and active have been recognized for MMP9.[11] The active form is important in the degradation of ECM and progression of tumors.[11, 22] In the present study, we used Elisa method which measures both the pro and active forms in serum.[11] Therefore, one reason for this difference between the case and control groups might have been the measurement of both forms rather than the active form alone.

Measuring the enzymatic activities of the MMP9 rather than measuring their concentration would be more valuable in implementing the experimental studies.[11]

The study of Zhang et al. states that MMP9 in tumor tissue might be expressed; however, its production is sometimes unstable and is maintained at nearly undetectable levels.[15]

One possible reason for this decreased level in patients might be the changes of MMP9 concentration with tumor progression. One of the limitations of the present study is that we looked for just one shot serum evaluation of MMP9 and multiple sessions of serum analysis were not performed. Therefore, further study can be conducted where multiple samples from the same patients can be collected at different time intervals; the changes in concentration of MMP9 during the progress of the disease can be assessed.

Another possible reason for decreased MMP9 level might be that all tissue MMP-9s were not released into to the serum, therefore serum level of MMP-9 could not show the tissue level of MMP-9. This will show that simultaneous evaluation of serum and tissue MMP-9 would be useful in the future researches.

In a study conducted by Lipari et al. no significant differences emerged in the expression of MMP2 and MMP9 in normal samples and benign salivary gland tumors.[23]

In the present study we measured serum level of MMP9 with Elisa technique, but Lipari et al. evaluated this marker on the tissue samples and used immunohistochemistry and RT-PCR technique.[23] They used RT-PCR to evaluate MMP-9 expression. As PCR does not provide the information about the level of gene product, their different results compared with our study might be related to the different methods used.

In this study, MMP9 levels were higher in patients with malignant tumor than benign group but the difference was not significant. This finding was in accordance with that found by Nagel et al. which exhibit no significant difference in MMP9 expression between malignant and benign tumors in the tissue samples.[7]

One possible reason for this finding in the present study is measuring MMP9 concentration rather than the enzymatic activity; hence, we recommend further studies with employing other methods to assess the active form of MMP-9. 

## Conclusion

Our result showed that serum level of MMP9 decreased in patients with salivary gland tumors which suggest that MMP9 may not have a potential role in development and pathogenesis of salivary gland tumor but this preliminary result may form the basis for future investigations to investigate the role of active MMP-9 in pathogenesis and progression of salivary gland tumor. 
